# Competing risk analysis of cardiovascular-specific mortality in typical carcinoid neoplasms of the lung: A SEER database analysis

**DOI:** 10.1097/MD.0000000000035104

**Published:** 2023-10-06

**Authors:** Hongquan Xing, Cong Wu, Dongdong Zhang, Xinyi Zhang

**Affiliations:** a Department of Respiratory Diseases, The Second Affiliated Hospital of Nanchang University, Nanchang, China; b Department of Pathology, The Second Affiliated Hospital of Nanchang University, Nanchang, China.

**Keywords:** cardiovascular mortality, competing risk analysis, SEER, typical carcinoid

## Abstract

Cardiovascular mortality (CVM) is a growing concern for cancer survivors. This study aimed to investigate the mortality patterns of individuals with typical carcinoid (TC) tumors, identify independent predictors of CVM, and compare these risk variables with those associated with TC deaths. The Surveillance, Epidemiology, and End Results (SEER) database from 2000 to 2019 was utilized for obtaining data on patients with TC. Standardized mortality rates were employed to evaluate the risk of CVM while multivariate competing risk models were used to determine the association between patient characteristics and the probability of CVM or TC-related deaths. Our findings show that TC patients had an increased risk of CVM, with an standardized mortality rates of 1.12 (95% CI:1.01–1.25). Furthermore, we discovered that age at diagnosis, marital status, year of diagnosis, SEER stage, site, year of diagnosis, surgery, radiotherapy, and chemotherapy all contributed independently to the risk of CVM in patients with TC, whereas age at diagnosis, sex, race, SEER stage, site, year of diagnosis, surgery, radiotherapy, and chemotherapy all contributed significantly to TC mortality. Compared to the general population in the United States, patients with TC are significantly more likely to acquire CVM. Timely introduction of cardioprotective treatments is critical for preventing CVM in patients with TC.

## 1. Introduction

Typical carcinoid (TC) tumors originate from neuroendocrine cells in the bronchopulmonary mucosa and account for approximately 1% of all lung tumors.^[1]^ Its prevalence in the United States and Europe ranges from 0.2 to 2/100,000.^[2]^ These tumors have become more common in recent decades, possibly due to improved diagnostic procedures or an increase in their occurrence.^[3]^

The natural progression of TC is often sluggish and indolent, with a 5-year overall survival (OS) with limited illness ranging from 87% to 100%.^[4]^ Furthermore, the 5-year survival rate of patients with advanced TC has increased to 60%.^[5]^ Other causes of death have become more common as life expectancy increases and cancer mortality decreases.^[6]^ A growing number of studies have shown that cardiovascular mortality (CVM) significantly affects the overall survival of long-term cancer survivors.^[7,8]^ Low, et al reported that CVM is a competing cause of death in almost 50% of patients with carcinoid tumors and remains a major competing cause of death even years after diagnosis.^[9]^

In addition, cardiac complications are well recognized in patients with carcinoid tumors and carcinoid syndrome.^[10]^ Approximately 50% of patients with carcinoid tumors develop carcinoid syndromes.^[11]^ 50% to 70% of patients with carcinoid syndrome develop carcinoid heart disease.^[12,13]^ Regarding the pathology of carcinoid tumors, the tumor releases vasoactive substances such as 5-hydroxytryptamine, prostaglandins, histamine, and tachykinins. These substances cause the deposition of plaque-like fibrous tissue in the endocardium, leading to valve dysfunction and eventual progression to heart failure.^[14]^ After heart failure occurs, the prognosis is severely affected, and the survival period is typically less than 1 year.^[15]^

Unfortunately, although several studies have aimed to quantify the risk of TC tumor-specific death or overall survival,^[16,17]^ few reports have investigated the risk factors for CVM in TC patients, thereby creating difficulties in the precise clinical management of these patients.

In the field of cardiac oncology, the Surveillance, Epidemiology, and End Results (SEER) database serves as a frequently employed resource for research purposes.^[18,19]^ This study aimed to estimate the risk of CVM in comparison to the general population, using the SEER database, and to identify TC patients at an increased risk of CVM. Our study may facilitate the development of more precise surveillance approaches and preventive interventions to mitigate the incidence of CVM in this patient population.

## 2. Methods

### 2.1. Data source

The SEER database contains information on approximately 28% of the population of the United States and provides follow-up information, demographic data, and clinical information on cancer patients. The National Center for Health Statistics updates statistics from this database annually.^[20]^ As part of our research, we chose 18 Registry Research Datasets (2000–2019, November 2021 submitted) from the SEER database (version 8.4.0.1). The standardized mortality ratio (SMR) was calculated using cases obtained from the SEER Research Plus Data, 17 Registries (excluding Alaska), and Nov 2021 Sub (2000–2019) for SMR.

### 2.2. Inclusion and exclusion criteria

**Figure 1.**
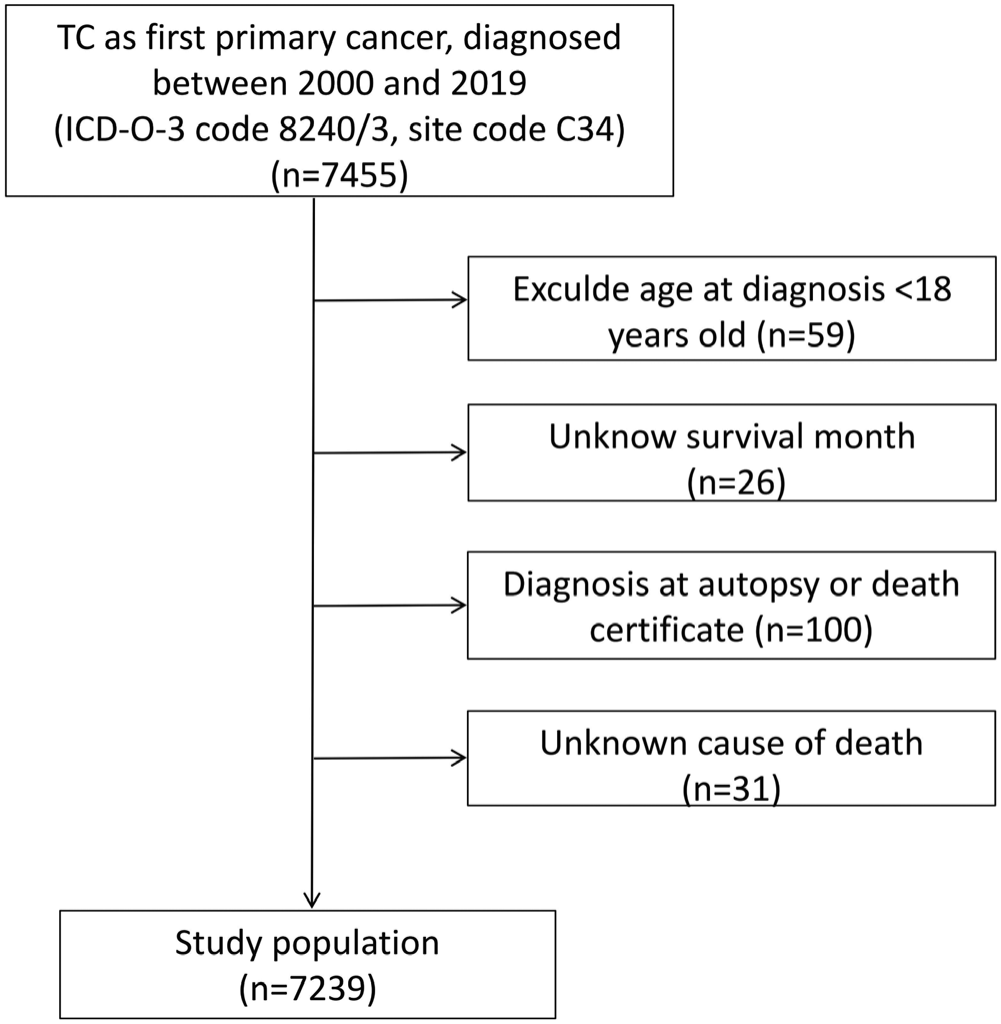
Flow chart of patient enrollment. TC = typical carcinoid, ICD-O-3 = International Classification of Diseases for Oncology, Third Edition.

Our criteria for evaluating cases were as follows: International Classification of Diseases for Oncology, Third Edition code:8240/3, site code: C34; The first primary tumor was TC with histological confirmation; and; Patients diagnosed from 2000 to 2019. The exclusion criteria were as follows: Age at diagnosis < 18 years; Unknown follow-up information; Autopsy diagnosis or death certificate, and; Unknown cause of death. The inclusion and exclusion criteria are presented in Figure 1.

Variables obtained from the SEER database included the year of diagnosis, age (18–54 years, 55–64 years, 65–74 years, ≥75 years), sex, race, marital status, subsite location (left lower lobe, left-upper lobe, right-upper lobe, right-middle/low lobe, unknown), summary stage, radiation recovery, chemotherapy, surgical operation, survival months, vital status recode, cause of death, and SEER cause-specific death classification. We divided the causes of death into 4 categories: TC death, CVM, other cancers, and other non-cancer causes. See Table S1, Supplemental Digital Content, http://links.lww.com/MD/K24, which illustrates the classifications about non-cancer deaths. CVM included 6 cause-specific deaths, and other non-cancer deaths included 8 cause-specific deaths.^[17,18]^ The follow-up time was defined as the span between the initial diagnosis of TC and the occurrence of death, the final follow-up, or the end of the follow-up period (December 31, 2019).

### 2.3. Statistical analysis

In this study, we compared the risk of CVM in TC patients to that in the general population in the United States and utilized SMR to report the results. The SMR was calculated by dividing the observed number of deaths by the expected number of deaths using the SEER Stat software. We used the Poisson exact method to determine the 95% confidence interval (CI) and corresponding p-values for SMR. We employed Fine and Gray competing risk regression analysis to account for the competing events of CVM and non-CVM deaths, and used the crude cumulative mortality function to estimate the probability of primary and competing events. Multivariate competitive risk survival analysis was conducted to identify independent predictors of CVM and TC mortality, and deep forest plots were used to compare these predictors.^[19]^ This analysis considered information about therapy and pathological and demographic characteristics. Empower Stats (www.empowerstats.com) and R (http://www.R-project.org) were utilized in all analyses. Statistical significance was defined as *P* ≤ .05.

## 3. Results

### 3.1. Patient characteristics

As shown in Table 1, our study included 7239 patients after adopting the inclusion criteria from 2000 to 2019, of whom 355 patients (4.90%) had CVM and 410 (5.66%) died due to primary cancer. The mean age of the patients with TC was 58 ± 15 years, and the median CVM was 64 months. Table 1 summarizes the baseline characteristics of all eligible patients. Among them, the ratio of women (n = 4973, 68.70%), white (n = 6466, 89.32%), and married (n = 4162, 57.49%) was very high. Most of the normal sites were found in the right-middle/low lobe (n = 2795, 38.61%), followed by the left lower lobe (n = 1447, 19.99%), and other regions. Based on the SEER stage, 4273 (59.03%) patients had localized tumors. In terms of treatment, surgery was performed on 5945 patients (82.12%), chemotherapy was administered to 252 patients (3.48%), and radiation therapy was administered to 266 patients (3.67%).

**Table 1:** Characteristics of patients with first primary typical carcinoid*.

Variables	N	Non-cancer deaths		Cancer deaths	
		CVM	Other non-cancer	Typical carcinoid	Other cancer
Total	7239	355 (4.90%)	554 (7.65%)	410 (5.66)%	184 (2.54)%
Years					
2000–2004	1257 (17.36%)	142 (40.00%)	212 (38.27%)	104 (25.37%)	64 (34.78%)
2005–2009	1503 (20.76%)	106 (29.86%)	163 (29.42%)	119 (29.02%)	50 (27.17%)
2010–2014	1804 (24.92%)	65 (18.31%)	129 (23.29%)	104 (25.37%)	37 (20.11%)
2015–2019	2675 (36.95%)	42 (11.83%)	50 (9.03%)	83 (20.24%)	33 (17.93%)
Age at diagnosis					
18–54	2536 (35.03%)	39 (10.99%)	73 (13.18%)	72 (17.56%)	39 (21.20%)
55–64	1754 (24.23%)	47 (13.24%)	100 (18.05%)	90 (21.95%)	34 (18.40%)
65–74	1863 (25.74%)	123 (34.65%)	181 (32.67%)	123 (30.00%)	56 (30.43%)
≥75	1086 (15.00%)	146 (41.13%)	200 (36.10%)	125 (30.49%)	55 (29.89%)
Sex					
Male	2266 (31.30%)	125 (35.21%)	181 (32.67%)	156 (38.05%)	86 (46.74%)
Female	4973 (68.70%)	230 (64.79%)	373 (67.33%)	254 (61.95%)	98 (53.26%)
Race					
White	6466 (89.32%)	324 (91.27%)	512 (92.42%)	352 (85.85%)	157 (85.33%)
Not white†	773 (10.68%)	31 (8.73%)	42 (7.58%)	58 (14.15%)	27 (14.67%)
Marital status					
Married	4162 (57.49%)	155 (43.66%)	276 (49.82%)	212 (51.71%)	99 (53.80%)
Not married	2706 (37.38%)	181 (50.99%)	252 (45.49%)	173 (42.20%)	76 (41.30%)
Unknown	371 (5.13%)	19 (5.35%)	26 (4.69%)	25 (6.10%)	9 (4.89%)
Subsite location					
Left lower lobe	1447 (19.99%)	92 (25.92%)	109 (19.68%)	61 (14.88%)	35 (19.02%)
Left-upper lobe	1110 (15.33%)	38 (10.70%)	72 (13.00%)	61 (14.88%)	20 (10.87%)
Right-upper lobe	975 (13.47%)	46 (12.96%)	83 (14.98%)	63 (15.37%)	28 (15.22%)
Right-middle/low lobe	2795 (38.61%)	131 (36.90%)	213 (38.45%)	137 (33.41%)	62 (33.70%)
Unknown	912 (12.60%)	48 (13.52%)	77 (13.90%)	88 (21.46%)	39 (21.20%)
SEER stage					
Local	4273 (59.03%)	143 (40.28%)	245 (44.22%)	83 (20.24%)	30 (16.30%)
Regional	1154 (15.94%)	57 (16.06%)	62 (11.19%)	83 (20.24%)	23 (12.50%)
Distant	689 (9.52%)	30 (8.45%)	59 (10.65%)	148 (36.10%)	75 (40.76%)
Unknown	1123 (15.51%)	125 (35.21%)	188 (33.94%)	96 (23.41%)	56 (30.43%)
Radiotherapy					
Yes	266 (3.67%)	345 (97.18%)	540 (97.47%)	322 (78.54%)	162 (88.04%)
No/unknown	6973 (96.33%)	10 (2.82%)	14 (2.53%)	88 (21.46%)	22 (11.96%)
Chemotherapy					
Yes	252 (3.48%)	347 (97.75%)	544 (98.19%)	308 (75.12%)	147 (79.89%)
No/unknown	6987 (96.52%)	8 (2.25%)	10 (1.81%)	102 (24.88%)	37 (20.11%)
Surgery					
Yes	5945 (82.12%)	118 (33.24%)	157 (28.34%)	243 (59.27%)	110 (59.78%)
No/unknown	1294 (17.88%)	237 (66.76%)	397 (71.66%)	167 (40.73%)	74 (40.22%)

CVM = cardiovascular- specific mortality.

* Database “SEER Research Plus Data, 17 Registries (excl AK), Nov 2021 Sub (2000–2019)” was used.

† Not white, Black, American Indian/AK Native, Asian/Pacific Islander, Unknown.

### 3.2. Comparison of CVM in study participants versus the general population

The SMR for CVM among TC patients was 1.12 (95% CI:1.01–1.25). Stratifying by different variables in the subgroup analysis, the patients were male, unmarried; aged 18 to 54 and 65 to 74 years at diagnosis, with latency periods of 0 to 1, 2 to 5 and 6 to 11 months; with a primary site in the left lower lobe, with a local stage, without chemotherapy, without radiotherapy, and without surgery, and had a significantly higher SMR than the general population (Table 2). The SMR of TC patients who died of CVM is shown in Table 3. The highest SMR was found in hypertension without heart disease (SMR, 7.06, 95% CI: 3.22–13.40), followed by cerebrovascular disease (SMR, 3.57, 95% CI: 2.67–4.67), and heart disease (SMR, 3.18, 95% CI: 2.82–3.58).

**Table 2:** Standardized mortality ratios of cardiovascular disease mortality among typical carcinoid patients*.

Characteristic		Observed deaths	Expected deaths	SMR	95% CI
Total		354	314.89	1.12	1.01–1.25
Years					
	2000–2004	142	113.77	1.25	1.05–1.47
	2005–2009	105	104.37	1.01	0.82–1.22
	2010–2014	65	68.04	0.96	0.74–1.22
	2015–2019	42	28.7	1.46	1.05–1.98
Latency (mo)					
	0–1	17	5.98	2.84	1.65–4.55
	2–5	19	11.41	1.67	1.00–2.60
	6–11	16	16.5	0.97	0.55–1.57
	12–59	118	114.38	1.03	0.85–1.24
	60–119	113	100.63	1.12	0.93–1.35
	120+	71	65.98	1.08	0.84–1.36
Age at diagnosis					
	18–54	39	21.25	1.84	1.30–2.51
	55–64	46	41.19	1.12	0.82–1.49
	65–74	123	90.14	1.36	1.13–1.63
	≥75	146	162.3	0.90	0.76–1.06
Sex					
	Male	125	91.18	1.37	1.14–1.63
	Female	229	223.71	1.02	0.90–1.17
Race					
	White	324	292.17	1.11	0.99–1.24
	Not white†	30	22.72	1.32	0.89–1.89
Marital status					
	Married	155	163.57	0.95	0.80–1.11
	Not married	180	138.11	1.30	1.12–1.51
	Unknown	19	13.21	1.44	0.87–.25
Subsite location					
	Left lower lobe	92	65.15	1.41	1.14–1.73
	Left-upper lobe	38	42.76	0.89	0.63–1.22
	Right-upper lobe	46	47.56	0.97	0.71–1.29
	Right-middle/low lobe	130	128.11	1.01	0.85–1.20
	Unknown	48	31.31	1.53	1.13–2.03
SEER stage					
	Local	143	158.81	0.90	0.76–1.06
	Regional	57	35	1.63	1.23–2.11
	Distant	30	25.19	1.19	0.80–1.70
	Unknown	124	95.88	1.29	1.08–1.54
Radiotherapy					
	Yes	10	6.95	1.44	0.69–2.65
	No/unknown	344	307.94	1.12	1.00–1.24
Chemotherapy					
	Yes	8	5.2	1.54	0.66–3.03
	No/unknown	346	309.69	1.12	1.00–1.24
Surgery					
	Yes	236	247.57	0.95	0.84–1.08
	No/unknown	118	67.32	1.75	1.45–2.10

CI = confidence interval, SMR = standard mortality ratio.

* Database “SEER Research Plus Data, 17 Registries (excl AK), Nov 2021 Sub (2000–2019) for SMRs” was used.

† Not white, Black, American Indian/AK Native, Asian/Pacific Islander, Unknown.

**Table 3:** The standardized mortality ratios of all causes of cardiovascular mortality in typical carcinoid patients*.

CVM	Observed deaths	Expected deaths	SMR	95% CI
Diseases of heart	283	88.89	3.18	2.82–3.58
Hypertension without heart disease	9	1.27	7.06	3.22-13.4
Cerebrovascular diseases	53	14.85	3.57	2.67–4.67
Atherosclerosis	1	1.08	0.93	0.01–5.16
Aortic aneurysm and dissection	4	1.97	2.03	0.55–5.19
Other diseases of arteries, arterioles, capillaries	4	2.37	1.69	0.45–4.33

CI = confidence interval, CVM = cardiovascular-specific mortality, SMR = standard mortality ratio.

* Database “SEER Research Plus Data, 17 Registries (excl AK), Nov 2021 Sub (2000–2019) for SMRs” was used.

### 3.3. Cumulative mortality of CVM

**Figure 2.**
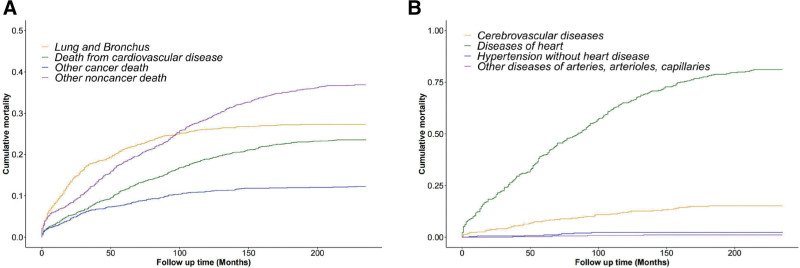
Cumulative mortality for all causes of death (A) and main causes of CVM (B) in TC patients. CVM = cardiovascular mortality, TC = typical carcinoid.

**Figure 3.**
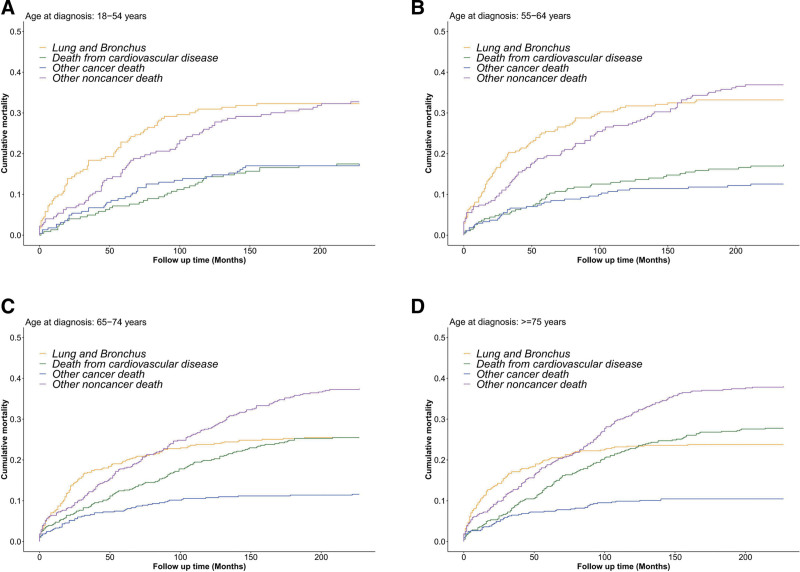
Cumulative mortality for all causes of death in TC patients stratified by age at diagnosis: (A) age between 18 and 54, (B) age between 55 and 64, (C) age between 65 and 74, and (D) age ≥ 75. TC = typical carcinoid.

The cumulative incidence function curves depicting mortality for all causes of death in TC patients using the Fine-Gray competing risk model are shown in Figure 2. During early follow-up, the highest cumulative mortality was caused by primary cancer. The incidence of CM from noncancerous disease exceeded that of primary cancer by approximately 100 months after diagnosis (Fig. 2A). After a 200-month follow-up, the cumulative incidence rates of cardiac and cerebrovascular diseases were 9.80% and 1.89%, respectively (Fig. 2B). Subgroup analyses stratified by age at diagnosis revealed that CVM exhibited a steady increase with advancing age at diagnosis (Fig. 3). Among patients aged ≥ 75 years, CM for cardiovascular disease surpassed CM for primary cancer as the second leading cause of death approximately 112 months after diagnosis (Fig. 3).

### 3.4. Univariate and multivariate analysis by fine-gray competing risk model in TC patients

We analyzed each of the factors listed above using the Fine-Gray univariate competing risk model. According to Table 4, sex, race, radiotherapy, and chemotherapy were not significantly correlated with CVM. However, years, marital status, age, site, SEER stage, and surgical treatment were significantly associated with CVM.

**Table 4:** Univariable Fine-Gray competing risk model analysis for CVM and TC death in patients with first primary typical carcinoid.

Variables		CVM		TC death	
		HR (95% CI)	*P* value	HR (95% CI)	*P* value
Years					
	2000–2004	Reference		Reference	
	2005–2009	0.592 (0.450,0.778)	<.001‡	0.923 (0.701,1.217)	.572
	2010–2014	0.262 (0.191,0.360)	<.001‡	0.728 (0.554,0.956)	.023*
	2015–2019	0.090 (0.063,0.131)	<.001‡	0.371 (0.276,0.499)	<.001‡
Age at diagnosis					
	18–54	Reference		Reference	
	55–64	1.742 (1.122,2.704)	.013*	1.771 (1.273,2.462)	<.001‡
	65–74	4.329 (3.020,6.204)	<.001‡	2.432 (1.791,3.304)	<.001‡
	≥75	9.744 (6.542,13.876)	<.001‡	4.381 (3.249,5.908)	<.001‡
Sex					
	Male	Reference		Reference	
	Female	0.872 (0.687,1.106)	.258	0.730 (0.592,0.889)	.003†
Race					
	White	Reference		Reference	
	Not white§	0.788 (0.537,1.157)	.224	1.499 (1.135,1.981)	.004†
Marital status					
	Married	Reference		Reference	
	Not married	1.789 (1.426,2.246)	<.001‡	1.304 (1.052,1.617)	.016*
	Unknown	1.268 (0.746,2.155)	.381	1.477 (0.965,2.259)	.072
Subsite location					
	Left lower lobe	Reference		Reference	
	Left-upper lobe	0.538 (0.370,0.785)	.001†	1.308 (0.911,1.879)	.146
	Right-upper lobe	0.726 (0.501,1.050)	.089	1.392 (0.960,2.018)	.081
	Right-middle/low lobe	0.771 (0.547,1.088)	.140	1.099 (0.800,1.508)	.561
	Unknown	0.761 (0.549,1.055)	.101	2.353 (1.690,3.276)	<.001‡
SEER stage					
	Local	Reference		Reference	
	Regional	1.507 (1.101,2.063)	.011*	3.880 (2.818,5.342)	<.001‡
	Distant	1.286 (0.850,1.944)	.234	13.773 (10.444,18.164)	<.001‡
	Unknown	3.883 (2.947,5.116)	<.001‡	4.753 (3.514,6.428)	<.001‡
Radiotherapy					
	No/unknown	Reference		Reference	
	Yes	0.697 (0.371,1.311)	.263	9.784 (7.601,12.595)	<.001‡
Chemotherapy					
	No/unknown	Reference		Reference	
	Yes	0.632 (0.309,1.292)	.208	13.295 (10.395,17.005)	<.001‡
Surgery					
	No/unknown	Reference		Reference	
	Yes	0.219 (0.174,0.275)	<.001‡	0.117 (0.094,0.146)	<.001‡

Statistically significant results are displayed with bold values. Statistical significance was indicated as.

CI = confidence interval, CVM = cardiovascular-specific mortality, HR = hazard ratio, TC = typical carcinoid.

* *P* value < .05.

† *P* value < .01.

‡ *P* value < .001.

§ Not white, Black, American Indian/AK Native, Asian/Pacific Islander, Unknown.

Subsequently, a multivariate competing risk model was employed to study the influence of these factors on CVM, and it was found that they remained significantly associated with this outcome. Specifically, the likelihood of CVM significantly increases with advancing age in patients with TC. Compared to patients aged 18 to 54 years, the hazard ratios (HRs) were 2.573 (95% CI: 1.538–4.304), 6.493 (95% CI: 4.253–9.913), and 12.634 (95% CI:7.980–20.003) for patients aged 55 to 64, 65 to 74 and ≥ 75 years, respectively. In unmarried patients, there was a greater chance of CVM, with an HRs of 1.416 (95% CI: 1.036–1.937). For primary sites of TC, the left lower lobe also had a higher risk of CVM than other primary sites (left-upper lobe vs left lower lobe, HR = 0.548, 95% CI: 0.329–0.912; right-upper lobe vs left lower lobe, HR = 0.488, 95% CI: 0.292–0.815; right-middle/low lobe vs left lower lobe, HR = 0.704, 95% CI:0.493–1.005). Patients diagnosed with distant disease had a significantly lower risk of developing CVM than those with localized disease (HR:0.457, 95% CI: 0.244–0.854). Furthermore, the study indicated that patients who underwent surgery had a lower risk of CVM (HR = 0.219, 95% CI: 0.174–0.275). After adjusting for the HRs for statistically significant variables established via univariate analysis, including age at diagnosis, marital status, years, site, and SEER stage, the adjusted risk ratio for resection was significantly reduced (in model 1, adjusted HR = 0.709, 95% CI: 0.512–0.982). The adjusted HR values remained consistent after further adjustments in Model 2, which included all baseline covariates (Tables 4 and 5).

**Table 5:** Multivariate competing-risks regression analysis of cardiovascular death

Variable		Unadjusted HR		Model 1^a^		Model 2^b^	
		HR (95% CI)	*P*	HR (95% CI)	*P*	HR (95% CI)	*P*
Surgery							
	No/unknown	Reference		Reference		Reference	
	Yes	0.219 (0.174, 0.275)	<0.001^***^	0.709 (0.512, 0.982)	0.038^*^	0.626 (0.445, 0.883)	0.008^**^

Statistical significance was indicated as *P < 0.05, **P < 0.01, or ***P < 0.001.

HRs = hazard ratios.

a Model 1: HRs were adjusted for statistically significant factors according to univariate analysis (years, age at diagnosis, marital status, subsite location, SEER stage and surgery).

b Model 2: HRs were adjusted for all factors in the baseline.

As shown in Table 4, sex, race, radiotherapy, chemotherapy, year, marital status, age, site, SEER stage, and surgical treatment were significantly associated with TC death. Using a multivariate competing risk model, we subsequently estimated the effects of these factors on TC death and found that age at diagnosis, years, SEER stage, chemotherapy, radiation, and surgery were significant risk factors for TC death. In particular, advanced age is associated with a higher risk of TC death. Compared to patients aged 18 to 54 years, the HRs were 1.710 (95% CI: 1.111–2.632), 1.939 (95% CI: 1.274–2.951), and 2.313 (95% CI: 1.525–3.508) for patients aged 55 to 64, 65 to 74 and ≥ 75 years, respectively. Patients with worse SEER stage had a significantly higher risk of TC death (Regional SEER stage vs Local SEER stage, HR = 2.778, 95% CI: 1.818–4.246; Distant SEER stage vs Local SEER stage, HR = 3.913, 95% CI: 2.631–5.818). Patients receiving chemotherapy and radiotherapy displayed a significant increase in the probability of CVM, with corresponding HRs of 2.651 (95% CI: 1.944–3.615) for chemotherapy versus those not receiving chemotherapy and 3.020 (95% CI: 2.171–4.200) for radiation therapy versus those not receiving radiation therapy. Conversely, patients who opted for surgery exhibited a reduced risk of total coronary death compared with those who did not, with an HR of 0.338 (95% CI: 0.248–0.461).

### 3.5. Forest plots of the risk factors and hazard ratios of CVM and TC death

**Figure 4.**
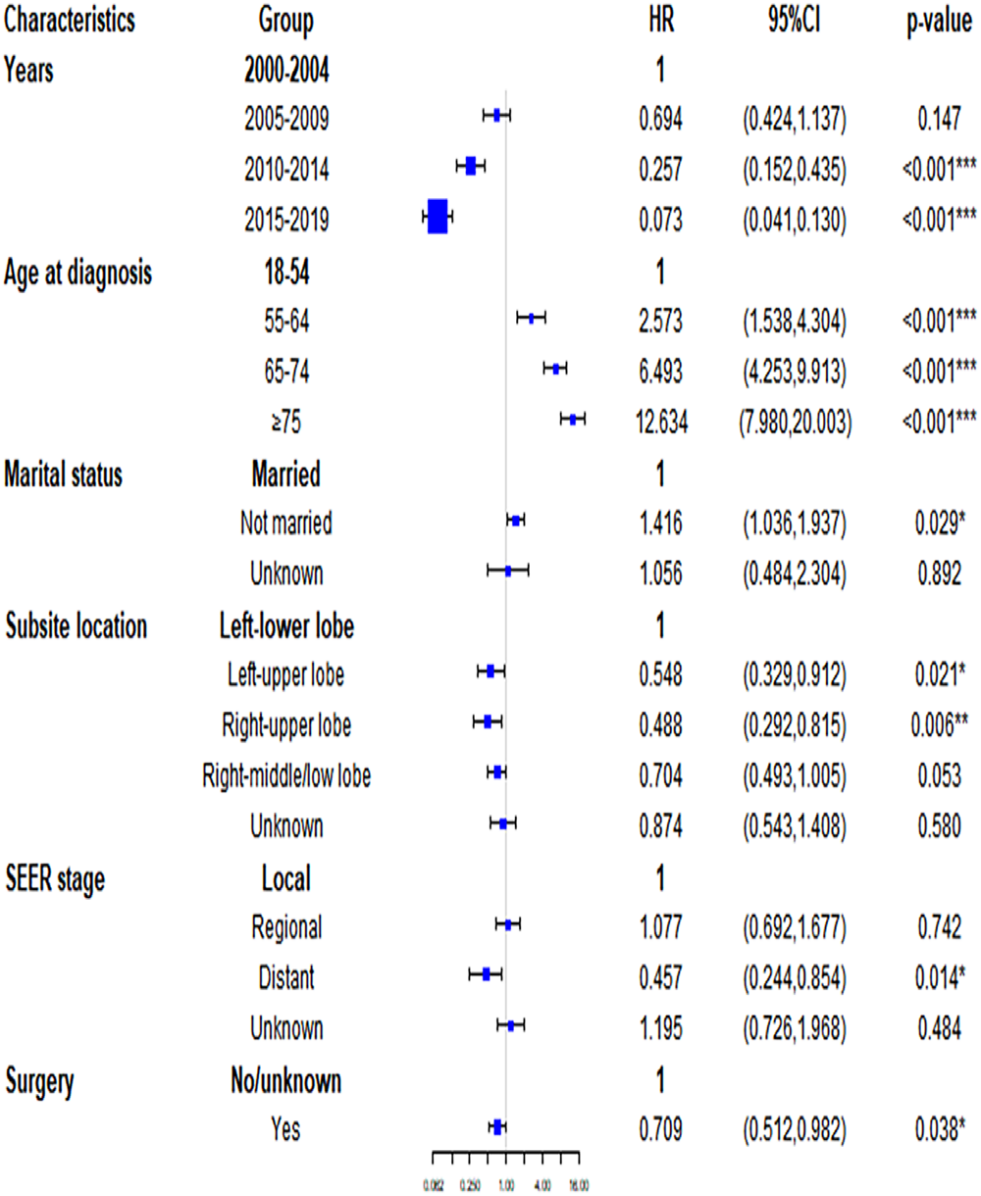
Forest plot of the risk factors associated with CVM. CI = confidence interval, CVM = cardiovascular mortality, HR = hazard ratio, SEER = Surveillance, Epidemiology, and End Results database.

**Figure 5.**
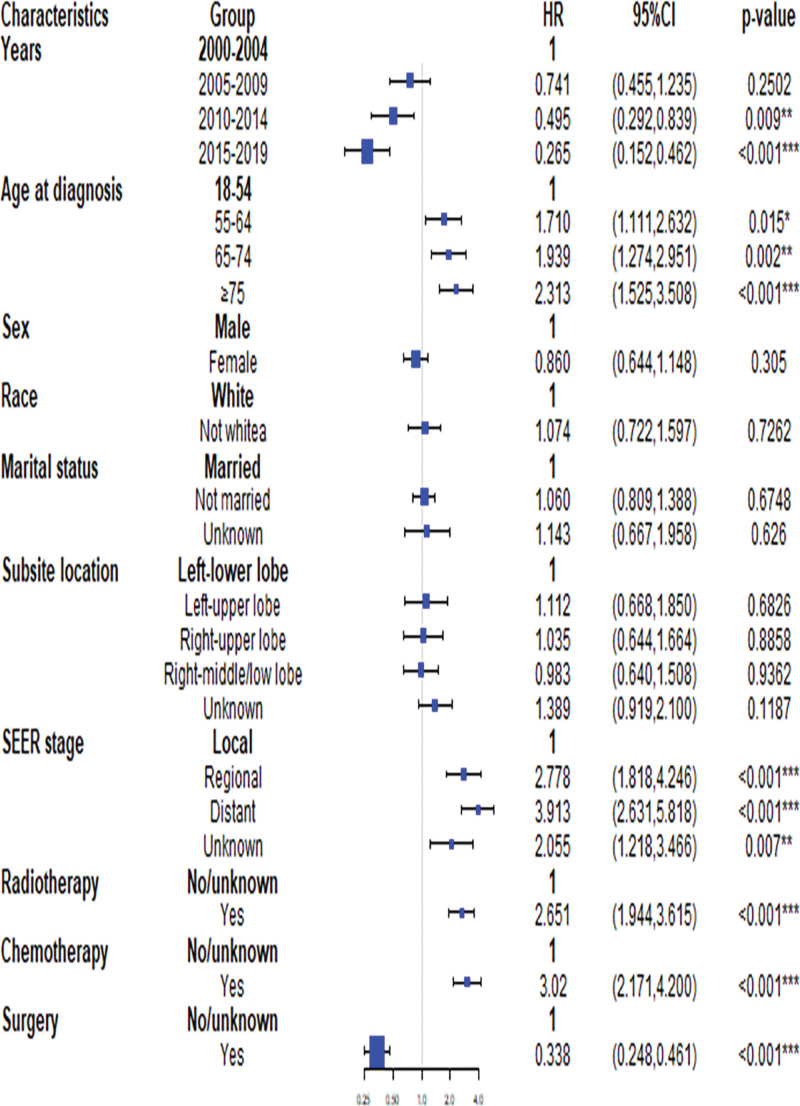
Forest plot of the risk factors associated with TC mortality. TC = typical carcinoid, SEER = surveillance, epidemiology, and end results database, HR = hazard ratio, CI = confidence interval.

Our findings from the multivariate competing risk analyses for CVM and TC death in TC patients are presented visually in Figures 4 and 5 using bent forest plots. Upon comparing the results, we identified differences in the risk factors associated with CVM and TC death. Notably, age was found to be a stronger risk factor for CVM, with marriage and primary site showing associations with CVM. In contrast, age did not exhibit a relatively high HR for TC death, and neither marriage nor the primary site was significantly associated with TC death. We further discovered that surgery was the most significant risk factor for TC death, while the SEER stage showed opposite HRs for TC death compared with CVM. HRs for radiotherapy and chemotherapy also differed between CVM and TC deaths.

## 4. Discussion

Despite existing research into various matters concerning TC patient survival, such as postoperative overall survival, TC-specific survival, and survival rates in metastatic TC,^[16,17,21]^ analyses of CVM within this population remain lacking. Furthermore, most earlier studies assessing overall survival have typically combined all outcomes into a single result, which may be inadequate for identifying TC patients facing different outcome risks. In this study, we analyzed the risk of CVM in TC patients compared with the general population based on the SEER database. Employing a competing risk model, we analyzed the risk elements associated with CVM and TC mortality and estimated their HRs, which were then visualized using a forest plot to compare these risk factors.

In this study, we found that the CVM risk of TC patients was 12% higher than that of the general US population (SMR: 1.12, 95% CI: 1.01–1.25). In addition, consistent with the results of a previous study conducted by Fang et al^[22]^, our analysis determined that TC patients are at the highest risk of CVM during the initial 2-month period following cancer diagnosis (SMR: 2.84; 95% CI: 1.65–4.55). This discovery may be due to the fact that cancer patients received aggressive treatment shortly after diagnosis and remained at high risk for a long time until their death.^[23]^ This finding could also be due to the fact that the patients who died early had the most severe cardiovascular comorbidities. Unfortunately, owing to the limitations of SEER, there is no information related to preexisting diseases before cancer diagnosis. However, these findings support the early involvement of cardiologists.

Some evidence supports the conclusion that tumor resection can slow the progression or prevent the development of carcinoid heart disease.^[24–26]^ However, because of the small sample size, these studies are limited, while our study included large and multicenter cases. Although this study could not consider the cardiovascular complications of the study subjects, the comparison also included populations with or without cardiovascular complications in the general population. SMR analysis has been widely used in similar studies^[27,28]^ to balance the impact of past comorbidities. Compared to the group of patients with tumors but without surgical treatment, we found that patients who underwent tumor resection surgery had a significantly reduced risk of CVM (no resection SMR:1.75; tumor resection SMR:0.95). Considering the effect of confounding variables, we further adjusted for potential confounding variables and noted a significant decrease in the odds of CVM development among individuals who underwent tumor resection, standing at approximately 30% lower than those who were not subjected to surgical intervention (in model 1, adjusted HR = 0.709; in model 2, adjusted HR = 0.626, both *P* < .05). Although we were unable to eliminate all potential confounding variables completely, we did our best to validate the study results. Similar to earlier reports,^[18,29]^ our investigation highlights a noteworthy decline in CVM susceptibility among cancer survivors who underwent surgery compared to their nonsurgical counterparts. Our results provide strong evidence indicating a link between CVM risk and tumor resection, further reinforcing the conclusion that resecting the primary tumor reduces the risk of CVM in cancer survivors.

With respect to the year of diagnosis, we found that the HRs of CVM and TC deaths in patients diagnosed with TC in 2000 to 2004, 2005 to 2009, 2010 to 2014 and 2015 to 2019 continued to decrease over time. The key to this finding may lie in advances in cardiovascular disease imaging techniques and biomarkers that have made early detection and intervention possible.^[30]^ In addition, patient prognosis has improved significantly with the development of a multidisciplinary approach and a significant increase in valve replacement survival rates.^[31,32]^

Our study determined that age is the primary risk factor contributing to CVM and TC mortality. Upon analysis, we noted an escalation in cumulative mortality associated with CVM among patients diagnosed at an older age, with CVM identified as the second most prevalent cause of death among those aged 75 years or older. Interestingly, the highest SMR for CVM was observed in patients aged 18 to 49 years (SMR: 1.84; 95% CI: 1.30–2.51), which is consistent with previous findings by Xie et al^[19]^ One possible reason is that the decreased physical function and overall poor health condition of elderly patients increases the risk of complications such as infections. Consequently, this may reduce their lifespan and prevent them from developing cardiovascular diseases.

Our results indicate that unmarried patients have a greater likelihood of experiencing CVM than married individuals, as reported in a previous breast cancer research.^[33]^ A plausible explanation exists for the greater likelihood of emotional support for married patients compared to unmarried patients, while positive social and psychological factors can potentially ameliorate the prognosis of cardiovascular disease.^[34]^ These findings suggest that unmarried patients with TC may require more psychological support and compassionate care for their management. Other studies have also shown that marriage helps improve cardiovascular and cancer prognosis.^[35–37]^ However, marriage was not significantly associated with TC-specific mortality.

Regarding the pathological characteristics of the patients, the probability of cardiac death was significantly higher in patients with lower left tumors, in agreement with the study by Jiang et al^[38]^ According to recent clinical studies, patients with NSCLC have a poorer prognosis if the primary site is not located in the upper lobe.^[39]^ However, cancer site did not exhibit a significant correlation with TC mortality. To date, the specific mechanism explaining this phenomenon is still unclear, but further clinical and laboratory research may provide more information about the relationship between the primary tumor site in TC patients, cardiovascular events, and TC mortality. Patients with worse SEER staging had a higher risk of death from TC and a lower risk of developing CVM than those with a limited stage. Possible reasons for this are that patients with advanced tumors may not live long enough to prevent them from developing and dying from cardiovascular disease.

Our study has some limitations. First, while we have effectively assessed the risk factors linked to CVM and TC death, certain information concerning CVM, including hyperlipidemia, diabetes, dyslipidemia, obesity, cardiovascular comorbidities, personal history of smoking, and alcohol consumption, was not accessible due to SEER’s limitations of SEER. Additionally, there was a small amount of missing data for certain parameters. Second, this study involved retrospective analysis of a broad US population database. Despite our stringent screening criteria, inherent limitations and potential for selection bias cannot be completely ruled out, as is the case with all retrospective studies. Nonetheless, the results of this study are relevant and may provide useful information for clinical management.

## 5. Conclusion

In summary, TC patients exhibit a significantly elevated risk of CVM compared to the general population. Age at diagnosis, marital status, tumor site, tumor stage, and surgery were associated with CVM, with differences in correlation compared to TC death. Evidence is provided for timely screening of CVM in patients with TC. Additional studies are necessary to establish a clinically useful prognostic model for CVM in TC patients. Furthermore, gaining a better understanding of the underlying mechanisms of CVM in patients with TC is imperative for the development of successful prevention and surveillance strategies.

## Author contributions

**Conceptualization:** Hongquan Xing, Xinyi Zhang.

**Data curation:** Hongquan Xing, Cong Wu, Dongdong Zhang, Xinyi Zhang.

**Formal analysis:** Hongquan Xing, Cong Wu, Dongdong Zhang, Xinyi Zhang.

**Funding acquisition:** Xinyi Zhang.

**Investigation:** Hongquan Xing, Cong Wu, Xinyi Zhang.

**Methodology:** Hongquan Xing, Cong Wu, Dongdong Zhang, Xinyi Zhang.

**Project administration:** Hongquan Xing, Xinyi Zhang.

**Resources:** Hongquan Xing, Cong Wu, Xinyi Zhang.

**Software:** Hongquan Xing, Cong Wu, Xinyi Zhang.

**Supervision:** Hongquan Xing, Cong Wu, Xinyi Zhang.

**Validation:** Hongquan Xing, Cong Wu, Xinyi Zhang.

**Visualization:** Hongquan Xing, Cong Wu, Xinyi Zhang.

**Writing – original draft:** Hongquan Xing, Cong Wu, Dongdong Zhang, Xinyi Zhang.

**Writing – review & editing:** Hongquan Xing, Cong Wu, Dongdong Zhang, Xinyi Zhang.

## Supplementary Material
