# Supplementary material for: Competing risk analysis of cardiovascular-specific mortality in typical carcinoid neoplasms of the lung: A SEER database analysis
Source: Medicine (Baltimore). 2023 Oct 6;102(40):e35104. doi: 10.1097/MD.0000000000035104 (PMC10553134; doi:10.1097/MD.0000000000035104)
Supplement: Supplementary file 1 [file medi-102-e35104-s001.docx]

| **Supplementary Table S1. The classifications about Non-cancer deaths** | | | | | | |
| --- | --- | --- | --- | --- | --- | --- |
| **Non-cancer deaths** | **Specific classifications** | | | | | |
| **Cardiocerebr ovascular  diseases** | Aortic  Aneurysm  and  Dissection | Atheroscler osis | Cerebrovasc ular  Diseases | Diseases  of Heart | Hypertension  without  Heart  Disease | Other Diseases of Arteries, Arterioles, Capillaries |
| **Infection** | Other  Infectious  and Parasitic  Diseases  including  HIV | Septicemia | Tuberculosis | Syphilis |  |  |
| **Diabetes  Mellitus** | Diabetes  Mellitus |  |  |  |  |  |
| **Alzheimer's  disease** | Alzheimer's  (ICD-9 and  10 only) |  |  |  |  |  |
| **Respiratory  diseases** | Chronic  Obstructive  Pulmonary  Disease and  Allied Cond | Pneumonia  and  Influenza |  |  |  |  |
| **Digestive  diseases** | Chronic  Liver  Disease and  Cirrhosis | Stomach  and  Duodenal  Ulcers |  |  |  |  |
| **Kidney  diseases** | Nephritis,  Nephrotic  Syndrome  and  Nephrosis |  |  |  |  |  |
| **Suicide,  accidents  and homicide** | Suicide,  accidents  and homicide | Homicide  and Legal  Intervention | Suicide and  Self Inflicted  Injury |  |  |  |
| **Other non neoplastic  diseases** | Certain  Conditions  Originating  in Perinatal  Period | Complicatio ns of  Pregnancy,  Childbirth,  Puerperium | Congenital  Anomalies | Other  Cause of  Death | Symptoms,  Signs and Ill Defined  Conditions |  |
